# Integrins and pulmonary fibrosis: Pathogenic roles and therapeutic opportunities

**DOI:** 10.17305/bb.2025.12545

**Published:** 2025-06-19

**Authors:** Zhangyang Bi, Guodong Zang, Xiaodong Wang, Li Tian, Wei Zhang

**Affiliations:** 1 Clinical Department of Integrated Traditional Chinese and Western Medicine, The First Clinical Medical College of Shandong University of Traditional Chinese Medicine, Jinan, China; 2 Department of Respiratory and Critical Care Medicine, Affiliated Hospital of Shandong University of Chinese Medicine, Jinan, China

**Keywords:** Integrin, pulmonary fibrosis, PF, targeted therapy

## Abstract

Characterized by the formation of fibrotic scars, pulmonary fibrosis (PF) involves a complex pathogenesis, limited treatment options, and a high mortality rate. Integrins—heterodimeric transmembrane proteins composed of α and β subunits—mediate extracellular matrix remodeling and regulate the physiological functions of epithelial, mesenchymal, and immune cells through “inside-out” and “outside-in” signaling pathways. These molecules play a critical role in the initiation and progression of PF. Due to their central regulatory functions, a range of integrin-targeted therapies has been developed. However, the complex pathophysiology of PF and the structural diversity of integrins pose significant challenges to targeted treatment. In this study, we systematically delineated the signaling networks mediated by the full spectrum of integrin family members and uncovered the molecular mechanisms by which they contribute to PF through immunoregulatory pathways. We also reviewed the development of integrin-based therapies from preclinical studies to clinical trials and discussed current priorities in clinical, basic, and translational research. These insights may provide new perspectives for the diagnosis and treatment of PF.

## Introduction

**Figure 1.**
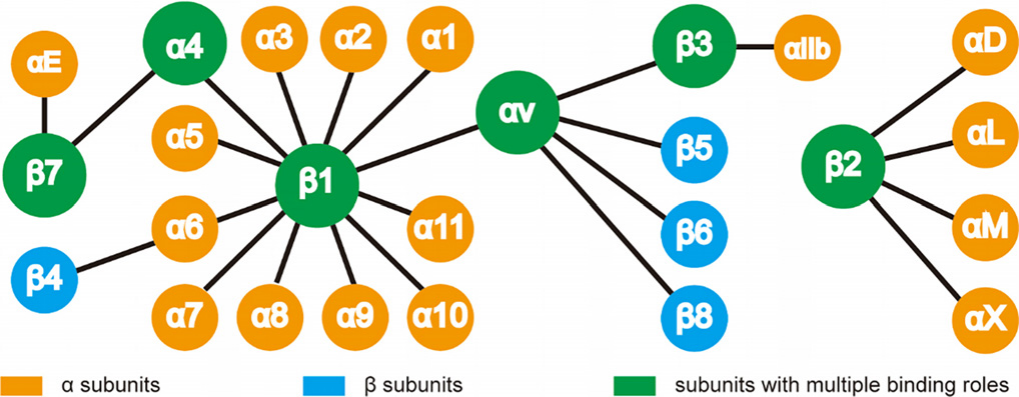
**The combination of integrin subunits forms integrin molecules.** The orange represents the α-subunit, the blue indicates the β-subunit, and the green denotes the subunit with multiple binding roles.

**Figure 2.**
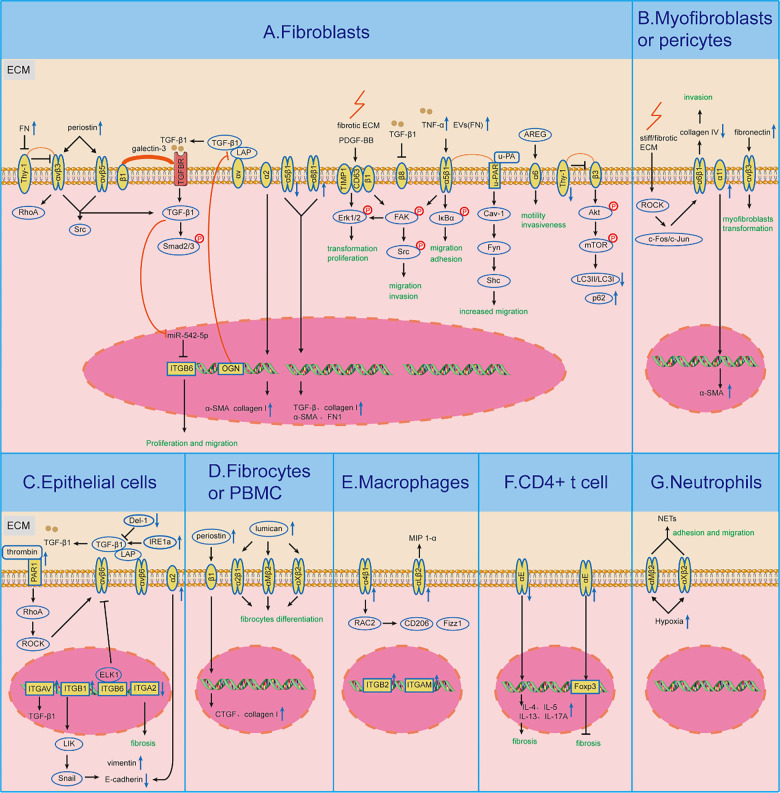
**Summary diagram illustrating the role of integrins in the seven specific cell types involved in the pathogenesis of pulmonary fibrosis.** The cell types are described as follows: (A) Lung fibroblast cells; (B) Lung myofibroblasts or pericytes; (C) Lung epithelial cells; (D) Fibrocytes or peripheral blood mononuclear cells; (E) Macrophages; (F) CD4+ T cells; and (G) Neutrophils. Arrows indicate the following: *↑* denotes promotion or activation; T and *↓* indicate reduction or inhibition; p represents phosphorylation; the lightning bolt icon denotes fibrotic ECM.

Pulmonary fibrosis (PF) represents the end stage of a diverse group of diffuse interstitial lung diseases, including nonspecific interstitial pneumonia, fibrotic hypersensitivity pneumonitis (fHP), connective tissue disease-associated interstitial lung disease (ILD), and idiopathic PF (IPF). PF is pathologically characterized by the progressive degradation of lung tissue architecture, often resulting in impaired lung function, compromised gas exchange, and, ultimately, respiratory failure and death. Studies indicate that patients with IPF have a 5-year mortality rate ranging from 30% to 50%, with a median survival of 2–3 years [1]. For patients with fHP, the reported median survival is approximately 7.1 years [2]. The interaction between cells and the extracellular matrix (ECM) plays a critical role in the initiation, progression, and prognosis of PF [3]. Integrins, which are heterodimeric transmembrane glycoproteins composed of α (120–185 kDa) and β (90–110 kDa) subunits, are central to this process. In mammals, more than 20 functionally distinct integrin molecules have been identified (Figure 1). As adhesion receptors, integrins are uniquely capable of bidirectional signaling across the plasma membrane, mediating essential interactions between cells and their surrounding microenvironment [4]. In outside-in signaling, integrins specifically bind extracellular ligands, triggering conformational changes that recruit and activate intracellular signaling molecules. This cascade regulates key cellular behaviors such as proliferation, differentiation, migration, and invasion. In inside-out signaling, intracellular activators bind to the cytoplasmic domains of integrins, causing conformational shifts that increase their affinity for extracellular ligands, promoting cell migration and altering the extracellular landscape. During the progression of PF, integrins mediate abnormal signaling among epithelial, mesenchymal, and immune cells, as well as with the ECM. This dysregulated signaling promotes the proliferation, activation, and migration of pathogenic cells, triggers the secretion of pro-fibrotic factors, and leads to excessive ECM deposition. Three primary regulatory pathways are implicated: the integrin–transforming growth factor β (TGF-β) axis, integrin-mediated mechanotransduction, and the integrin–immunity axis. For example, αv and α2β1 integrins specifically activate TGF-β signaling. Meanwhile, integrins such as αvβ3, αvβ5, α2β1, α4β1, and α5β1 facilitate epithelial–mesenchymal transition (EMT), as well as fibroblast migration, invasion, and differentiation by transmitting mechanical cues from the ECM to cells. On immune cells, integrins including α4β1, αLβ2, αMβ2, αXβ2, and αE amplify pro-inflammatory and pro-fibrotic responses via pathways such as RhoA/Rho kinase (ROCK). Given their central role in PF pathogenesis, targeting integrin-mediated signaling represents a promising therapeutic strategy to disrupt the fibrotic cascade and slow disease progression. Several integrin-targeted therapeutics are currently being evaluated in clinical trials for safety, tolerability, and efficacy. Numerous other lead compounds and candidates are undergoing preclinical development in academic and industry settings, contributing to a growing body of meaningful data. In this review, we searched PubMed using keyword combinations such as (“integrin” OR “αvβ6” OR “αvβ1,” etc.) AND (“pulmonary fibrosis” OR “IPF” OR “lung fibrosis”), focusing on publications from January 2014 to March 2025 (one month prior to submission). We included original research (both preclinical and clinical), high-quality review articles, and clinical trial registry entries (https://clinicaltrials.gov/). Our review provides a comprehensive overview of integrin-mediated pathogenesis in PF (Table 1). Specifically, we summarize integrin-related signaling across seven key cell types (Figure 2), describe the mechanisms of action of integrin inhibitors, and highlight promising candidates currently in clinical trials (Tables 2 and 3). Finally, we discuss major challenges and future directions in exploring integrin-targeted therapies for PF.

## Role of integrin-dependent signaling axis in PF

### Integrin/TGF-β signaling pathway in PF

The integrin αv subunit can pair with β1, β3, β5, β6, or β8 subunits to form various integrin heterodimers with distinct functions, many of which play key roles in fibrosis development. Studies have shown that αv integrins activate latent TGF-β—a classical pro-fibrotic cytokine—thereby promoting PF in multiple experimental models [5]. Galectin-3 has been found to bind both αvβ1 integrin and TGFβRII in a glycosylation-dependent manner, facilitating their spatial co-localization. This interaction enhances TGF-β1 activation and downstream signaling [6]. In lung fibroblasts, αvβ3 and αvβ5 integrins interact with periostin to activate the TGF-β/Smad3 signaling pathway, leading to upregulation of lung fibrosis-associated molecules such as SERPINE1, CTGF, IGFBP3, and IL-11 [7]. Integrin αvβ6 also engages TGF-β, contributing to both physiological and pathological responses. TGF-β1 regulates ITGB6 gene expression via the Smad pathway, which in turn influences αvβ6 integrin expression [8]. Activation of the αvβ6/TGF-β axis is implicated in TGF-α-induced pleural fibrosis and PF triggered by influenza infection [9, 10]. Additional research has shown that OGN knockdown or elevated levels of developmental endothelial locus-1 (Del-1) can inhibit integrin αv—particularly αvβ6—from binding to the latency-associated peptide (LAP), thereby suppressing TGF-β/Smad signaling [11, 12]. After lung injury, IRE1α is upregulated and modulates damage-associated transient progenitor cells (DATPs), enhancing αvβ6 expression and promoting fibrosis via TGF-β activation [13]. Thrombin has also been shown to drive fibrosis through the PAR1/αvβ6/TGF-β pathway. *In vitro* studies reveal that PAR1 triggers αvβ6-mediated TGF-β activation via RhoA and ROCK, identifying a potential therapeutic target in PF [14, 15]. In canine IPF, the ITGB8 gene is significantly downregulated, and TGF-β stimulation leads to decreased integrin β8 expression in MRC-5 cells [16]. These findings suggest that TGF-β may contribute to IPF progression in dogs by modulating integrin β8 expression. Researchers have also explored the heterogeneity of mesenchymal stromal cells and their roles in PF. Sciurba et al. [17] demonstrated that ITGAV promotes type 17/TGF-β-driven PF, as evidenced by reduced collagen deposition in bleomycin-induced ITGAV flox/flox (Pdgfrb-Cre^+^) mice. Yi et al. [18] reported decreased TGF-β1 activity, reduced p-Smad2/3 expression, and less radiographic lung fibrosis in ITGAV loxP/loxP; Pdgfrb-Cre mice following radiation exposure. Interestingly, deletion of αv integrin in α-smooth muscle actin (α-SMA)-positive cells did not protect against bleomycin-induced PF, while PDGFRβ-Cre-mediated deletion did. These findings suggest that α-SMA-positive cells are a subset of PDGFRβ-positive cells and are not the primary mediators of αv integrin–dependent TGF-β activation in PF [19].

**Table 1:** Potential mechanisms of integrin-induced PF

**Integrin subunits and molecules**	**Models**	***In vivo*/*in vitro***	**Main mechanisms**	**References**
αv	Cigarette smoke extract-induced and αv-silenced AECs	*In vitro*	Activated TGF-β1*↓*	[5]
αv	OGN-knockdown lung fibroblasts treated with or without TGF-β	*In vitro*	The binding of integrin αv to LAP*↓*, p-Smad2*↓*	[11]
αv	BLM-induced ITGAV *^flox/flox^* (Pdgfrb- cre+) mice	*In vivo*	HYP*↓*	[17, 19]
αv	ITGAV^lox*P*/lox*P*^; Pdgfrb-Cre mice receiving radiation	*In vivo*	Activated TGF-β1*↓*, p-Smad2/3*↓*	[18]
β1	BLM-induced mice	*In vivo*	β1*↑*	[46]
β1	PQ-induced-PF rats	*In vivo*	β1*↑*	[47]
β1	Elk1-knockout mice	*In vivo*	β1*↓*, spontaneous fibrosis*↑*	[48]
β1	BLM-induced mice, accompanied by loss of periostin in fibrocytes; periostin -/- fibrocytes	*In vivo* and *in vitro*	β1, CTGF, collagen I*↓*	[43]
β1	SiO_2_-induced and ITGB1-knockout BEAS-2B cells	*In vitro*	E-cadherin*↑*, vimentin*↓*, ILK, Snail*↓*	[49]
β1	MWCNT-induced PF in mice; lung fibroblasts	*In vivo* and *in vitro*	TIMP1/CD63/integrin β1*↑*, pErk1/2*↑*, lung fibroblast proliferation*↑*	[50]
β1	PDGF-BB induced lung fibroblasts with integrin β 1 blocking antibody	*In vitro*	Fak activation*↓*, fibroblast migration on FN*↓*	[20]
β1	Fibroblasts in fibrotic ECM	*In vitro*	Integrin-β_1_/FAK/ERK1/2*↑*, fibroblasts transforming into myofibroblasts	[21]
β3	ABCG1-deficient MWCNT mice	*In vivo*	MFG-E8, β3 in BALF cells*↑*	[34]
β3	LPS-induced β3-specific shRNAs in lung fibroblasts	*In vitro*	Activation of the PI3K-Akt-mTOR*↓*, LC3II/LC3I*↓*, p62*↑*	[67]
β3	Integrin β3-deficient MV mice	*In vivo*	PKM2, LDHA, lactate, collagen deposition*↓*	[68]
α1	Mice induced by adenoviral vector and BLM	*In vivo*	α1*↑*, fibrosis*↑*	[51]
α2	IPF patients	*In vivo*	α2*↑*	[52]
α2	DDR2-deficient mice; lung fibroblasts lacking Col1a1	*In vivo* and *in vitro*	α2*↑*, fibrosis*↓*	[53]
α2	TC-I-15-treated lung fibroblasts	*In vitro*	Collagen I and α-SMA*↓*	[53]
α2	Floxed integrin α2 mice crossed with Col1a2-creERT mice	*In vivo*	Fibrosis*↓*	[55]
α2	Floxed integrin α2 mice crossed with SPC-rtTA/tetO-Cre mice	*In vivo*	Fibrosis*↑*	[55]
α2	MTX-A549 cells	*In vitro*	α2*↑*, mRNA of EMT-related genes*↑*	[54]
α2, αMβ2 and αXβ2	Lumican-induced PBMC pretreated with α2, αM, αX, and β2 inhibitors	*In vitro*	Lumican-induced fibrocytes differentiation*↓*	[44]
α3	ITGA3 missense mutation	*In vivo*	Fibrosis*↑*	[56]
α4	LPS-induced mice	*In vivo*	Integrin α4β1*↑*	[33]
α4	BLM-treated α4Y991A mice	*In vivo*	Rac*↓*, M2 macrophage markers (e.g., YM1, Fizz1, CD206) *↓*	[38]
α5	Components in the supernatant secreted by IPF fibroblasts affect normal fibroblasts	*In vitro*	ITGA5, pIκBα↑, adhesion and migration *↑*	[57]
α5	Fibroblasts were incubated with EVs	*In vitro*	Fibroblast invasion*↑*, activation of FAK and Src*↑*	[22]
α5	IPF-HLFs	*In vitro*	uPAR*↑*, integrin α5*↑*, caveolin-Fyn-Shc*↑*in lipid rafts, fibroblast migration*↑*	[23]
α5	SPCs	*In vitro*	Fn1, Col1a1, ITGA5*↑*	[58]
α5	ITGA5-silenced IPF-HLFs	*In vitro*	Transformation of fibroblasts to myofibroblasts, FN1, TGF-β, α-SMA and collagen I*↑*	[59]
α6	Human IPF lung myofibroblasts in the sclerotic matrix	*In vitro*	ROCK*↑*, c-Fos/c-Jun transcription complex*↑*, α6 integrin*↑*, collagen IV*↓*	[24]
α6	BLM-induced PF in mice; HLF and MLF	*In vivo* and *in vitro*	CD11c+ AREG*↑*, integrin α6*↑*, lung fibroblast motility and invasiveness*↑*	[62]
α6	Silica dust-induced PF mice and TGF-β1-stimulated fibroblasts	*In vivo* and *in vitro*	miR-542-5p*↓*, ITGA6 *↑*, proliferation and migration of fibroblasts*↑*, lung fibrosis*↑*	[63]
α8	IPF patients	*In vivo*	CD248 ^low^ ITGA8 ^high^ fibroblast-like cells in the elastic fiber-rich connective tissue	[60]
α8	ITGA5 silenced fibroblasts	*In vitro*	Transformation of fibroblasts to myofibroblasts, ITGA8 and ITGAV*↑*	[59]
α10	BLM-induced MIF knockdown in rats	*In vivo*	α10*↓*, fibrosis*↓*	[64]
α11	IPF patients	*In vivo*	α11*↑*	[52]
α11	IPF patients	*In vivo*	α11+ α-SMA myofibroblasts*↑*	[65]
αMβ2	Human interstitial lung lesions following neococcal pneumonia	*In vivo*	sITGaM and sITGb2*↑*	[36]
αM and αX	Neutrophils under hypoxic conditions	*In vitro*	αM and αX*↑*, NETs*↑*	[39]
αLβ2 and αMβ2	HFD-fed mice	*In vivo*	ITGAM, ITGAL, ITGB2L, ITGB2, NAIP6 and NAIP5*↑*	[35]
αvβ1	HLFs	*In vitro*	Galectin-3 binds to β1 integrin, p-Smad2*↑*	[6]
αvβ3	BLM-induced mice	*In vivo*	αvβ3*↑*	[66]
αvβ3	Periostin or integrin silencing in lung fibroblasts	*In vitro*	Lung fibroblast proliferation*↓*	[27]
αvβ3 and αvβ5	Knockdown of integrin αv/β3/β5 in lung fibroblasts treated with TGF-β	*In vitro*	SERPINE1, CTGF, IGFBP3, and IL11*↓*	[7]
αvβ3	BLM mice lacking Thy-1	*In vivo*	αvβ3 in αSMA^+^ myofibroblasts*↑*	[28]
αvβ3 and αvβ5	PDGF-BB stimulated fibroblasts	*In vitro*	Src activation, Src interacted with integrative αvβ3, fibroblasts migration	[31]
αvβ3	Thy-1 neg fibroblasts	*In vitro*	RhoA activity*↑*, collagen matrix contraction*↑*	[30]
αvβ3	BLM-treated mice; CD146+ MACS-enriched primary cells seeded on fibronectin	*In vivo* and *in vitro*	Pericytes α-SMA*↑*, αvβ3*↑*	[26]
β6	TGF-β1 stimulation of iHBECs	*In vitro*	Time-dependent increase in ITGB6 mRNA	[8]
αvβ6	TGF-α-induced PF in mice	*In vivo*	αvβ6*↑*, TGF-β ↑	[10]
αvβ6	Influenza-infected mice	*In vivo*	αvβ6*↑*, p-smad2/3*↑*	[9]
αvβ6	Elk1-deficient BLM-induced mice; Elk1 siRNA IHBECs	*In vivo* and *in vitro*	ITGB6*↑*	[69]
αvβ6	Mice treated with Del-1 following BLM induction; HSAEpC cells incubated with inactive TGF-β were treated with Del-1	*In vivo* and *in vitro*	αvβ6*↓*, active TGF-β↓	[12]
αvβ6	Mice exposed to BLM and treated with the IRE1α kinase inhibitor KRA8	*In vivo*	Krt7 and ITGB6 double-positive cells *↓*, local TGF-β signaling and fibrosis*↓*	[13]
αvβ6	SFLLRN-stimulated immortomouse lung epithelial (IMLE) cells with transformed mink lung reporter (TML) cells + αvβ6 blocking antibody; SFLLRN-stimulated IMLE cells treated with the Rho kinase inhibitor Y-27632	*In vitro*	PAR1, αvβ6, TGF-β↓	[14]
αvβ8	West highland white terrier with IPF; TGF-β stimulated MRC-5 cells	*In vivo* and *in vitro*	ITGB8*↓*	[16]
αE	Mice exposed to aspergillus antigen	*In vivo*	CD69^hi^CD103^lo^ CD4+ TRM cells: IL5 and IL13*↑*; CD69^hi^CD103^hi^ Foxp3+ Treg cells: ITGAE, Foxp3*↑*	[40]

Abbreviations: TGF-β1: Transforming growth factor-β1; AEC: Alveolar epithelial cell; PF: Pulmonary fibrosis; MWCNT: Multiwalled carbon nanotube; TIMP1: Tissue inhibitor of metalloproteinase 1; PDGF-BB: Platelet-derived growth factor BB; FN: Fibronectin; ECM: Extracellular matrix; PI3K-Akt-mTOR: Phosphatidylinositol 3-kinase/protein kinase B/mammalian target of rapamycin; PKM2: Pyruvate kinase M2; LDHA: Lactate dehydrogenase A; IPF: Idiopathic pulmonary fibrosis; α-SMA: α-smooth muscle actin; EMT: Epithelial–mesenchymal transition; FAK: Focal adhesion kinase; SPC: Stromal progenitor cell.

### Integrin-mediated mechanotransduction in PF

The mechanism of integrin-mediated mechanotransduction in PF involves multiple levels of cellular signaling regulation. In PF, various integrins, particularly those containing the αv subunit, connect to the actin cytoskeleton by recognizing the RGD sequence in the LAP. This integrin-mediated mechanical tension acts on the LAP–TGF-β complex, inducing a conformational change that leads to the rupture of the disulfide bond between LAP and TGF-β. As a result, active TGF-β is released, initiating downstream signaling pathways. When lung tissue is damaged, ECM components such as collagen and fibronectin (FN) increase, leading to heightened tissue stiffness. Integrins, functioning as transmembrane receptors, link the ECM to the intracellular cytoskeleton, enabling the sensing of mechanical signals (e.g., ECM stiffness and tensile forces) and their conversion into biochemical cues. Integrin-mediated mechanotransduction pathways—primarily involving focal adhesion kinase (FAK), Src family kinases, and Rho GTPases—promote fibroblast migration, invasion, and transformation. This process is further amplified by increased ECM deposition, creating a vicious cycle. Upon stimulation with platelet-derived growth factor BB (PDGF-BB), FAK directly binds to integrin β1, promoting fibroblast migration toward FN. The integrins α5β1 and α4β1 are the primary mediators of FAK-dependent fibroblast migration [20]. The integrin β1/FAK/ERK1/2 signaling pathway facilitates the differentiation of fibroblasts into myofibroblasts within the fibrotic ECM microenvironment [21]. Senescent fibroblasts and those derived from patients with IPF (IPF-HLFs) secrete more extracellular vesicles (EVs), which are enriched in FN. These EVs interact with α5β1 integrins on fibroblasts, activating pro-invasive signaling pathways involving FAK and Src family kinases [22]. Notably, IPF-HLFs overexpress the urokinase-type plasminogen activator receptor (uPAR), which increases their migratory capacity. Binding of uPAR to its receptor binding domain shifts integrin signaling from the lipid raft–independent FAK pathway to the lipid raft–dependent caveolin–Fyn–Shc pathway. This signaling switch represents a novel mechanism underlying the enhanced migratory ability of fibrotic fibroblasts in IPF patients [23]. As a mechanosensitive molecule, α6 integrin regulates the invasive behavior of myofibroblasts and fibroblasts in response to matrix stiffening. A sclerotic ECM activates the c-Fos/c-Jun transcriptional complex via a ROCK-dependent mechanism, leading to increased α6 integrin expression, MMP-2–mediated hydrolysis of basement membrane collagen IV, and enhanced myofibroblast invasion [24]. Increased matrix stiffness and cellular stretching also activate integrin β3, which plays a critical role in vascular remodeling, as shown by computational modeling of pulmonary artery adventitial fibroblasts (PAAFs) [25]. During fibrosis, αvβ3 integrin promotes fibroblast activation and mechanotransduction by binding to provisional matrix components such as FN [26]. Moreover, periostin–integrin αvβ3 interactions play a key role in lung fibroblast proliferation [27]. Thy-1, a cell surface glycoprotein, can physically interact with inactive αvβ3 integrins, reducing their adhesion to FN. Studies have shown that the lung environment in Thy-1–deficient mice supports persistent αvβ3 integrin activation in fibroblasts, contributing to the sustained pro-fibrotic myofibroblast phenotype *in vivo* [28, 29]. Under normal conditions, integrins bind high-affinity ligands and induce Rho signaling and rigidity sensing. Deletion of Thy-1 results in rapid aggregation and upregulation of c-Src signaling downstream of αvβ3 integrin, leading to stiffness-insensitive RhoA activity, fibroblast activation, and collagen matrix contraction [30]. Additionally, Src kinase primarily interacts with αvβ5 and αvβ3 integrins to regulate fibroblast migration [31].

**Table 2:** Integrin inhibitors entering clinical studies

**Name (sponsor)**	**Modality**	**Delivery route**	**Integrin** **targets**	**Highest human dose reported/dose**	**Clinical Trials** **gov identifiers**	**Safety and efficacy**	**Study** **status^a^**	**Population or** **indication**
GSK3008348 (GlaxoSmithKline )	Small molecule	Topical inhalation	αvβ6	1 to 3000 ug	NCT02612051; NCT03069989;	Well tolerated	Phase I (terminated)	IPF and healthy volunteers
BG00011 (Biogen)	Humanized mAb	s.c.	αvβ6	56 mg weekly	NCT03573505	Toxicity observed	Phase II (terminated)	IPF
IDL-2965 (Indalo Therapeutics)	Small molecule	Oral	pan-αv	/	NCT03949530	/	Terminated	IPF
PLN-74809 (Pliant Therapeutics)	Small molecule	Oral	αvβ6, αvβ1	40 mg daily for 7 days [102]	NCT04396756	Good tolerability	Phase II Recruiting	IPF

Abbreviation: IPF: Idiopathic pulmonary fibrosis.

**Table 3:** The potential mechanism of integrin-based therapies in PF

**Integrin inhibitor**	**Targeted integrins**	**Models**	***In vivo*/*in vitro***	**Main mechanisms**	**References**
Cilengitide	Pan-αv	MRC-5 cells and mice after radiation	*In vitro* and *in vivo*	αv, active TGF-β1, Smad2/3 and p-Smad2/3*↓*, collagen and α-SMA protein*↓*	[18]
Cilengitide	Pan-αv	LPS-induced lung fibroblasts	*In vitro*	β3*↓*, PI3K-Akt-mTOR pathway activation*↓*, autophagy inhibition*↓*	[67]
Cilengitide	Pan-αv	MV mice	*In vivo*	PKM2, LDHA, lactate, collagen deposition*↓*	[68]
CWHM12	Pan-αv	TGF-β induced PF in PCLS	*In vitro*	αv*↓*, Aspn, Col1a1, Csrp2, Fap, Fbln2, Fbn2, Has2, Pappa and Wisp1*↓*	[79]
CWHM12	Pan-αv	Mechanically stretched fibrotic lung tissues	*In vitro*	αv*↓*, TGF-β1*↓*, p-Smad2/3*↓*	[80]
MK-0429	Pan-αv	BLM-induced mice	*In vivo*	Fibrosis*↓*	[81]
Ab-31	Pan-αv	IPF lung fibroblasts	*In vitro*	α-SMA*↓*	[81]
C8	αVβ1	BLM-induced mice	*In vivo*	Collagen*↓*	[82]
CP4715	αvβ3	Lung fibroblasts from IPF patients	*in vitro*	Down-regulation of the expression of proliferation- and cell cycle-related genes	[27]
CP4715	αvβ3	BLM-induced mice	*In vivo*	Attenuated PF and Smad3 activation	[7]
Cyclo(-RGDfK)	αvβ3	Myh11 profile-positive cells cultured on fibronectin-coated Petri dishes	*In vitro*	Inhibition of pericyte-to-myofibroblast transformation	[26]
B6_BP_dslf	αvβ6	Fibrotic lung-like organs	*In vitro*	α-SMA and fibronectin*↓*	[84]
Lovastatin	αLβ2	BLM-induced mice	*In vivo*	Inflammation, collagen deposition*↓*	[85]
α-αMβ2 and CBP-α-αMβ2	αMβ2	BLM-induced mice; Monocyte-derived myofbroblasts	*In vivo* and *in vitro*	collagen deposition*↓*, de-diferentiate mouse myofbroblasts	[86]
BTT 3033	α2β1	Lumican-induced PBMC	*In vitro*	Lumican-induced fibrocytes differentiation*↓*	[44]
TC-I 15	α2β1	TGF-β1 stimulated fibroblasts	*In vitro*	collagen I and α-SMA*↓*	[53]
E7820	α2β1	In MTX-treated A549 cells	*In vitro*	α-SMA*↓*	[54]
α-α3 and CBP-α-α3	α3β1	BLM-induced mice; Monocyte-derived myofibroblasts	*In vivo* and *in vitro*	Collagen deposition*↓*, de-differentiate mouse myofibroblasts	[86]
ATN-161	α5β1	O-PMs stimulated A549cells	*In vitro*	Fibronectin and vimentin*↓*, E-cadherin*↑*	[89]
ATN-161	α5β1	PQ-induced PF in rats	*In vivo*	Fibrosis*↓*	[21]
Echistatin	β1	Silicosis rats	*In vivo*	Snail, AKT and β-catenin*↓*, EMT*↓*	[90]
Pirfenidone	αMβ2	Co-culture of macrophages and lung fibroblasts	*In vitro*	M2 polarization and its adhesion, α-SMA, active TGF-β levels*↓*	[37]
Triptolide	β1	BLM-induced mice	*In vivo*	Integrin β1-FAK activation-mediated nuclear translocation of YAP1*↓*	[46]
IL-32	/	MRC-5 cells treated with TGF-β	*In vitro*	FAK and paxillin activation*↓*	[92]
yASCs	αv	BLM-induced PF in aged mice	*In vivo*	αv*↓*, collagen, MMP-2 activity and AKT phosphorylation, Caspase-9, TGF-β, TNF-α, VEGF-A*↓*, ros*↓*, nrf2*↑*	[93]
PD29	αvβ3 and αE	BLM-induced PF in rats	*In vivo*	αvβ3 and αE*↓*, TGF-β1/Smad3*↓*	[94]

Abbreviations: TGF-β1: Transforming growth factor-β; PI3K-Akt-mTOR: Phosphatidylinositol 3-kinase/protein kinase B/mammalian target of rapamycin; PKM2: Pyruvate kinase M2; LDHA: Lactate dehydrogenase A; α-SMA: α-smooth muscle actin; PF: Pulmonary fibrosis.

### Integrin-mediated inflammation and fibrosis in immune cells

The inflammatory response plays a crucial role in the early stages of PF [32]. Mice that received intratracheally administered LPS exhibited increased expression of very late antigen-4 (VLA-4, α4β1) in their lungs. Moreover, VLA-4 expression was significantly correlated with several inflammatory markers, including nitric oxide synthase-2 (NOS-2), interleukin-1β (IL-1β), and tumor necrosis factor-α (TNF-α) [33]. In mice deficient in the myeloid ATP-binding cassette cholesterol transporter (ABCG1) and exposed to multiwalled carbon nanotubes (MWCNTs), increased expression of MFG-E8 and integrin β3 indicated enhanced efferocytosis. This response can intensify lung inflammation and perpetuate the production of pro-fibrotic cytokines such as TGF-β [34]. In high-fat diet (HFD)-fed mice, the expression of integrin-inflammasome pathway genes (ITGAM, ITGAL, ITGB2L, ITGB2, NAIP6, and NAIP5) was significantly upregulated in lung tissue. Conversely, deletion of p16 downregulated multiple integrin pathway genes (e.g., ITGA4, ITGA7, ITGAL, ITGAM, ITGAX, ITGB2, and ITGB2L), suggesting that p16 may influence PF progression by modulating the integrin-inflammasome signaling pathway [35]. Integrins also contribute to the abnormal secretion of pro-fibrotic mediators and promote pathological ECM deposition by regulating various signaling pathways in immune cells such as macrophages, neutrophils, and T lymphocytes. Studies have shown that long-term pulmonary complications in post-COVID-19 patients are associated with elevated concentrations of soluble integrin subunits sITGaM and sITGb2 [36]. Database analyses revealed increased expression of RHOA, ITGB2, and ITGAM in alveolar macrophages, indicating mechanical activation of these cells within the extensively remodeled fibrotic microenvironment in human IPF lungs [37]. In BLM-induced PF models, α4 integrin mutant mice displayed reduced fibrosis and impaired M2 macrophage differentiation. Integrin α4β1 appears to promote the alternative (M2) activation of macrophages, which adopt a pro-fibrotic phenotype via Rac2 activation [38]. Additionally, hypoxia was shown to increase the expression of αM and αX integrin subunits in neutrophils, stimulate neutrophil extracellular trap (NET) formation, and contribute to a pro-fibrotic environment. The dose-dependent release of NETs triggered by αMβ2-specific agonists strongly supports integrin activation as a mechanistic link between hypoxia and NET generation. The co-localization of NETs with mesenchymal remodeling regions suggests that persistent NET release may directly exacerbate fibrosis [39]. Repeated exposure to fungal antigens led researchers to identify two distinct subpopulations of CD4+ TRM cells with opposing roles in PF. CD4+ TRM cells characterized by CD69hiCD103lo promoted lung fibrosis by producing effector cytokines such as IL-4, IL-5, IL-13, and IL-17A. In contrast, CD4+ regulatory T cells (Tregs) marked by CD69hiCD103hi expressed genes like Foxp3, which suppressed the fibrotic response [40]. Fibrocytes, circulating mesenchymal precursor cells derived from hematopoietic stem cells, can be recruited to fibrotic regions to produce ECM components [41]. Periostin, a matrix protein secreted by fibrocytes, mediates cell–ECM interactions [42]. In periostin-deficient mice, β1 integrin levels in fibrocytes (CD45+Col1+) were significantly reduced, and lung fibrosis was attenuated following BLM treatment. Periostin may activate fibroblasts via β1 integrin signaling, operating in a paracrine or autocrine fashion to induce the release of CTGF and other pro-fibrotic mediators and to stimulate collagen I synthesis [43]. Additionally, Pilling et al. demonstrated that TNF-α-stimulated fibroblasts secrete lumican, which promotes fibrocyte differentiation. The integrins α2β1, αMβ2, and αXβ2 are essential for lumican-induced differentiation of fibrocytes [44].

## The role of integrin molecules in PF

### Integrin α1β1–α11β1

The integrin subunit β1 can form heterodimers with integrin subunits α1–α11 and αv, all of which contribute to various fibrogenic functions in the PF process [45]. Integrin β1 was found to be significantly expressed in the fibrotic foci of animal models, with upregulation observed at multiple time points (2 and 4 weeks) [46, 47]. Cairns et al. [48] reported that ELK1-deficient mice developed spontaneous lung fibrosis by one year of age, which was associated with a marked decrease in ITGB1 mRNA levels and increased collagen deposition. Li et al. [49] found that knockdown of ITGB1 in BEAS-2B cells, combined with silica exposure, reduced expression of ILK/Snail pathway proteins and enhanced EMT. In lung fibroblasts, MWCNTs induced the expression of tissue inhibitor of metalloproteinase 1 (TIMP1), which formed a complex with CD63 and integrin β1. This complex activated the ERK1/2 pathway, promoting cell proliferation and providing insight into the molecular mechanisms underlying pro-fibrotic responses [50].

#### Integrin α1β1

Altered expression of integrin α1, a collagen receptor, may influence cellular interactions with the ECM. Both adenoviral vectors and BLM have been shown to significantly upregulate integrin α1 mRNA expression, contributing to lung fibrosis, inflammation, and tissue damage in mice [51].

#### Integrin α2β1

There was a substantial two-fold increase in ITGA2 expression in IPF samples, indicating a strong correlation between integrin α2 and PF [52]. Studies have shown that mice with fibroblast-specific deletion of α2 integrins are protected against fibrosis. Further research indicates that collagen I enhances TGF-β-mediated activation of collagen synthesis (COL1A1, ACTA2) and promotes myofibroblast activation via the α2β1 integrin in fibroblasts. Interestingly, collagen I/DDR2 signaling negatively regulates α2 integrin expression, suggesting that α2 may act as a compensatory receptor to sustain fibrosis in the absence of DDR2 [53]. In alveolar epithelial cells (AECs), α2 integrin appears to have a bidirectional role in regulating PF. Treatment with methotrexate (MTX) significantly increased both mRNA and cell surface expression of ITGA2 in A549 cells, along with the expression of genes associated with EMT [54]. However, mice with AEC-specific deletion of α2 integrins showed increased lung injury and a more severe fibrotic response [55]. Mechanistic studies suggest that collagen I inhibits caspase-3/7 activation and apoptosis in AECs via the α2β1 integrin pathway. Collectively, these findings indicate that the role of α2 integrins is highly context-dependent, influenced by factors such as cell type, microenvironment, stromal composition, and disease stage. Variability in downstream signaling pathways may lead to seemingly contradictory phenotypes.

#### Integrin α3β1

ILNEB syndrome includes ILD, nephrotic syndrome, and epidermolysis bullosa. Two patients carrying novel ITGA3 missense mutations in exons 3 and 6 presented with severe PF and skin manifestations but did not exhibit nephrotic symptoms. Both patients survived to the ages of 9 and 13 years, respectively [56].

#### Integrin α5β1 and α8β1

ITGA5 is highly expressed in IPF fibroblasts and is particularly enriched in fibroblast foci. TNF-α secreted by IPF fibroblasts increases ITGA5 expression in normal fibroblasts and activates the NF-κB pathway, enhancing their adhesion and migration capacity on FN [57]. Integrin α5 expression is also dynamic during mesenchymal cell differentiation. As stromal progenitor cells (SPCs) differentiate into myofibroblasts, genes such as FN1, Col1a1, and ITGA5 are upregulated; however, the proportion of ITGA5-expressing cells decreases with cell maturation [58]. Silencing ITGA5 in IPF-derived human lung fibroblasts (IPF-HLFs) results in reduced proliferation and migration, increased cell death, and promotes fibroblast-to-myofibroblast transformation. This transition is marked by elevated levels of ITGA8, FN1, TGF-β, α-SMA, and collagen I [59]. ITGA8 is mainly localized to fibrotic regions characterized by dense collagen deposition and abundant elastic fibers [59, 60]. However, in BLM-induced PDGFRβ-Cre;ITGA8flox/flox mice, deletion of ITGA8 did not significantly worsen fibrosis, suggesting that ITGA8 expression in PDGFRβ+ cells does not play a major biological role in lung fibrosis [61].

#### Integrin α6β1

Amphiregulin (AREG), a ligand of the EGFR, plays a critical role in tissue repair and fibrosis. In bone marrow-derived CD11c^+^ cells, recombinant AREG significantly upregulated the expression of α6 integrins on lung fibroblasts and enhanced their motility and invasiveness [62]. In both silica dust-induced PFmouse models and TGF-β1-stimulated fibroblasts, the expression of miR-542-5p was downregulated, while ITGA6 mRNA levels were markedly increased. Further investigation revealed that miR-542-5p directly targets the 3′-UTR of ITGA6, thereby inhibiting its expression and suppressing fibroblast proliferation and migration [63].

#### Integrin α10β1

Integrin α10 mRNA levels were significantly elevated in rats with BLM-induced PF. Inhibition of macrophage migration inhibitory factor (MIF) reduced the expression of ITGA10, suggesting that ITGA10 may serve as a potential therapeutic target for PF [64].

#### Integrin α11β1

The expression level of ITGA11 was significantly upregulated in samples from patients with IPF [52]. Further studies demonstrated that ITGA11 expression was also significantly elevated and co-localized with α-SMA–positive myofibroblasts in lung tissues from IPF patients [65].

### Integrin αvβ3 and αvβ6

#### Integrin αvβ3

The β3 subunit can bind to either the αv or αIIb subunit to form the αvβ3 and αIIbβ3 integrins. Integrin αvβ3 is significantly elevated in various subtypes of human ILD and in BLM-induced PF in mice [66]. *In vitro* experiments have shown that LPS inhibits autophagy in lung fibroblasts and promotes fibrosis by reducing the binding of Thy-1 to integrin β3 and activating the phosphatidylinositol 3-kinase/protein kinase B/mammalian target of rapamycin (PI3K-Akt-mTOR) pathway [67]. Downregulation of integrin β3 attenuates mechanical ventilation (MV)-induced aerobic glycolysis and subsequent PF, accompanied by decreased expression of pyruvate kinase M2 (PKM2) and lactate dehydrogenase A (LDHA) in lung tissue, as well as reduced lactate levels in BALF [68]. Interestingly, the binding of αvβ3 integrin to FN induces pericyte differentiation into a myofibroblast phenotype in BLM-treated mice [26].

#### Integrin αvβ6

αvβ6 is predominantly expressed in epithelial cells. Dysregulation of Elk1 has been observed in the epithelium of patients with IPF. *In vitro* experiments have demonstrated that Elk1 protein binding to the ITGB6 promoter inhibits transcription of the ITGB6 gene, resulting in reduced expression of integrin αvβ6 [69].

## Integrin-based therapies

**Figure 3.**
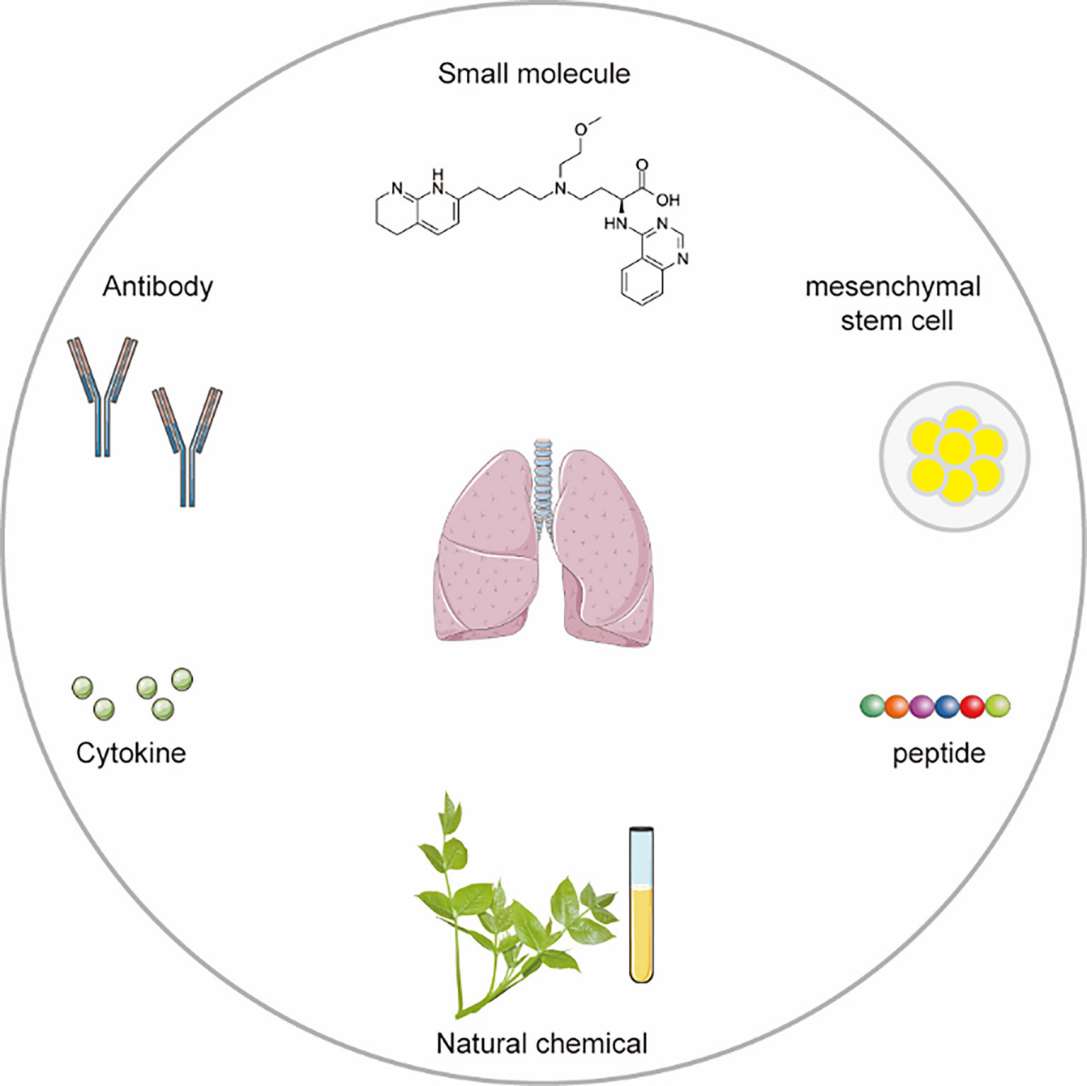
**Diverse modalities in integrin-focused therapy.** Current integrin-based therapies include small molecule, antibody, cytokine, natural chemical, peptide, and mesenchymal stem cell.

Therapeutic agents targeting integrins have been actively developed due to their diversity and potential for targeted treatment. To date, seven integrin-targeting drugs have been successfully marketed [70]. Although none are currently approved specifically for PF, the promising therapeutic outcomes observed in both clinical and preclinical studies support the potential of integrin-based therapies for PF. A range of integrin-targeted therapeuticsincluding small molecules, antibodies, synthetic mimetic peptides, and natural compounds—have been investigated and developed (Figure 3).

### Clinical studies of targeted integrin therapy

GSK3008348 is the first inhaled αvβ6 integrin inhibitor developed for the treatment of IPF. In human lung epithelial cells, GSK3008348 induces rapid internalization and lysosomal degradation of the αvβ6 integrin. It binds to αvβ6 with high affinity and effectively reduces downstream pro-fibrotic TGFβ signaling to normal levels in human IPF lungs, demonstrating good tolerability. Positive effects and safety were observed in a Phase Ib clinical study. However, GlaxoSmithKline discontinued development of GSK3008348 in 2018 following the Phase II clinical trial (NCT03069989), which failed to meet its efficacy endpoints. The decision likely also reflected a broader assessment of the drug’s commercial viability [71]. BG0001, an αvβ6-targeting monoclonal antibody, demonstrated antifibrotic activity in murine models. However, in a Phase IIb clinical study, the least squares mean change in forced vital capacity (FVC) was −0.097 L in the BG0001 group vs −0.056 L in the placebo group (*P* ═ 0.268). Additionally, a greater proportion of patients in the BG0001 group exhibited PF exacerbation on chest CT (44.4% vs 18.2%), along with more serious adverse events [72]. These effects may stem from the antibody’s prolonged half-life and high target affinity, potentially leading to excessive suppression of TGFβ1 and triggering acute exacerbations. Development of BG0001 was discontinued in early 2019 due to these safety concerns [73]. Development of IDL-2965 (NCT03949530), a pan-αv integrin inhibitor, was also halted in 2021 due to operational challenges and concerning non-clinical data [74, 75]. Bexotegrast (PLN-74809) is an oral small-molecule inhibitor targeting both αvβ6 and αvβ1 integrins. In studies using precision-cut lung slices from IPF patients, PLN-74809 significantly reduced type I collagen gene expression and TGF-β signaling [76]. In 2022, Pliant Therapeutics, Inc. reported encouraging results from the INTEGRIS-IPF Phase IIa trial, showing a modest reduction in FVC decline compared to placebo. Biomarker analysis (ITGB6 and PRO-C3) indicated a dose-dependent response. No drug-related serious adverse events or deaths were observed. PLN-74809 is currently considered the most promising αv integrin-targeting candidate in clinical development for IPF.

### Preclinical studies on integrin-based therapy

#### Targeted integrin therapy

**pan-αv**

Cilengitide is a potent integrin antagonist. Its IC50 values against the integrins αvβ3, αvβ5, and α5β1 are 0.61, 8.4, and 14.9 nM, respectively [77]. *In vivo*, cilengitide inhibited radiation-induced excess collagen and α-SMA production, and *in vitro*, it reduced the adhesion of lung fibroblasts to FN and hyaluronan by inhibiting the integrin αv/TGF-β1/Smad2/3 signaling pathway [18]. Additionally, cilengitide suppressed autophagy and inhibited LPS-induced activation of the PI3K-Akt-mTOR pathway in lung fibroblasts [67]. In MV mice, cilengitide reduced levels of PKM2, LDHA, and lactate, along with collagen deposition [68]. CWHM-12 is another potent inhibitor of αv integrins, specifically targeting αvβ8, αvβ3, αvβ6, and αvβ1, with IC50 values of 0.2, 0.8, 1.5, and 1.8 nM, respectively [78]. In a rat PCLS fibrosis model, CWHM-12 significantly suppressed the expression of fibrotic genes and the secretion of proteins such as Col1a1 and Wisp1 [79]. It also attenuated mechanical stretch-induced activation of TGF-β1 and phosphorylation of Smad2/3 in fibrotic lung strips over a range of inhibitory concentrations [80]. Zhang et al. identified integrin antibodies MK-0429 and Ab-31, which exhibit cross-reactivity in both human and mouse models and demonstrate significant anti-fibrotic effects. In mice, MK-0429 significantly inhibited the progression of BLM-induced PF. In lung fibroblasts derived from patients with IPF, Ab-31 markedly reduced α-SMA expression. MK-0429 strongly inhibited human integrins αvβ1, αvβ3, αvβ5, αvβ6, αvβ8, and α5β1, with IC50 values of 0.46, 0.15, 9.9, 3.8, 58.3, and 17.3 nM, respectively. Ab-31 also significantly inhibited mouse integrin-mediated cell adhesion for αvβ1, αvβ3, and αvβ5, with IC50 values of 1.5, 1.0, and 5.6 nM, respectively [81].

**αvβ1, αvβ3 and αvβ6**

Reed et al. [82] developed a potent and highly specific small-molecule inhibitor of αvβ1, known as C8, which significantly reduced collagen deposition in mice with BLM-induced PF. CP4715, a strong inhibitor of integrin αvβ3, effectively suppressed the proliferation of IPF fibroblasts [27]. Additionally, CP4715 reduced Smad3 activation and attenuated fibrosis in BLM-induced mouse models [7]. Cyclo(-RGDfK), a potent and selective αvβ3 integrin inhibitor with an IC50 of 2.25 nM [83], blocked the transition of pericytes to myofibroblasts and reduced α-SMA expression in FN-coated Myh11 lineage-positive cells [26]. Roy et al. [84] designed B6_BP_dslf to selectively inhibit αvβ6-mediated TGF-β activation, achieving IC50 values of 1.84 nM for αvβ6 and 32.8 nM for TGF-β. In a human lung-like organ model, B6_BP_dslf significantly reduced pro-fibrotic markers, including α-SMA and FN, demonstrating its anti-fibrotic potential.

**αLβ2 and αMβ2**

Leukocyte functional antigen-1 (LFA-1), an integrin expressed on the surface of leukocytes, interacts with hyaluronic acid receptors such as CD44 and TLR2, playing a crucial role in the regulation of inflammatory cytokine expression. Lovastatin uniquely binds to LFA-1 and significantly inhibits the expression of MIP-1α and other inflammatory cytokines induced by LMW HA in mouse alveolar macrophages. It also reduces fibrosis in a BLM-induced lung injury model [85]. Moreover, blocking antibodies targeting integrin αM (α-αM) and αMβ2 (α-αMβ2) promoted the dedifferentiation of monocytes into myofibroblasts and significantly reduced the secretion of profibrotic factors by these cells. When decorin’s collagen-binding peptide (CBP) was conjugated to these antibodies—creating CBP-α-αM and CBP-α-αMβ2—their accumulation in fibrotic lungs increased, thereby enhancing their antifibrotic efficacy and inhibiting the progression of lung fibrosis [86].

**α2β1, α3β1, α5β1 and α6β1**

BTT3033, an integrin α2 inhibitor, significantly inhibited lumican-induced fibrocyte differentiation [44]. Under specific experimental conditions, TC-I 15 inhibited α2β1 integrin with an IC_5__0_ of 0.4 µM for GLOGEN peptides and 26.8 µM for GFOGER peptides [87]. Treatment with TC-I15 also reduced the expression of collagen I and α-SMA in fibroblasts [53]. Similarly, the ITGA2 inhibitor E7820 inhibited MTX-induced EMT-associated phenotypic changes, including alterations in cellular morphology and decreased α-SMA expression [54]. Integrin α3 plays a critical role in the differentiation of monocytes into myofibroblasts. Like αMβ2, integrin α3 was shown to reverse myofibroblast differentiation and reduce the secretion of profibrotic factors [86]. The integrin α5β1 antagonist ATN-161—a short, five-amino-acid peptide derived from the synergistic region of FN—has a reported IC_5__0_ of 4.2 µM [88]. Administration of ATN-161 reduced the expression of FN and ETS-1, thereby inhibiting EMT progression in A549 cells treated with organic solvent–soluble PMs (OPMs) [89]. Moreover, ATN-161 attenuated paraquat (PQ)-induced PF and improved survival in rats [21]. Finally, echistatin, an integrin β1 inhibitor, was found to block EMT and lung fibrosis by interfering with various stages of the integrin β1/ILK/PI3K signaling pathway [90].

**Non-specific therapy based on integrins**

In macrophage–lung fibroblast co-culture microtissues, inhibition of ROCK2 and integrin αMβ2 by PFD prevented macrophage mechanical activation and reduced their ability to adhere, align, and spread. This, in turn, affected M2 polarization and pro-fibrotic activity [37]. Triptolide, a diterpenoid compound derived from the traditional Chinese medicine Tripterygium wilfordii Hook.f., was shown to inhibit integrin β1 expression, prevent FAK phosphorylation, block YAP1 translocation from the cytoplasm to the nucleus, and attenuate fibrosis progression [46]. Artemisinins, known for their potential clinical use in modulating immune responses and reducing inflammation, inhibited nuclear factor-κB (NF-κB) activity by targeting receptor-coupled signaling pathways—including β3 integrin—and downregulated numerous NF-κB-regulated genes such as cytokines, chemokines, and immune receptors [91]. IL-32γ reduced fibrosis marker expression by inhibiting the integrin–FAK–paxillin signaling axis [92]. In aged mice treated with young donor adipose-derived mesenchymal stem cells (yASCs), αv-integrin mRNA expression in the lungs was significantly reduced, leading to decreased lung fibrosis [93]. PD29, a 29-amino acid peptide with high biological activity and low toxicity, may prevent the onset and progression of PF through antiangiogenic effects, inhibition of matrix metalloproteinase activity, and suppression of integrin signaling. Specifically, PD29 significantly reduced expression of integrins αvβ3 and αE, thereby inhibiting PF development in rats with BLM-induced fibrosis [94].

## Discussion

PF is a multifactorial, end-stage lung disease associated with ILD, characterized by pathological features such as fibroblast proliferation and ECM deposition. These changes lead to the destruction of lung architecture and substantial loss of function, posing a serious global health concern. In investigating the underlying mechanisms, we found that integrin alterations in PF are influenced by temporal dynamics, spatial context (cellular microenvironment), and cell type, resulting in distinct functional outcomes. Notably, α4β1 integrins exhibit time-dependent dual roles: they promote inflammation in the early stages of PF and fibrosis in later stages. During fibroblast-to-myofibroblast transition, we observed a functional switch between integrin α5β1 and α8β1, highlighting spatial specificity in PF progression. Expression of α2β1 integrins in PF is modulated by ECM ligand type (laminin vs collagen I) and by cell identity (epithelial vs. fibroblast). The spatiotemporal behavior of α2β1 may reflect preferential regulation of distinct downstream signaling pathways in different model systems. Additionally, compensatory and alternative signaling within the integrin family contributes to the complexity of PF. Both αv and α8 integrins are upregulated in PDGFRβ-positive cells. However, knockdown of αv significantly inhibited PF progression, while α8 knockdown had a milder effect, suggesting that ITGAV plays a more prominent pro-fibrotic role than ITGA8 in these cells. Notably, deletion of αv integrins using α-SMA-Cre did not confer protection against BLM-induced PF, whereas deletion via PDGFRβ-Cre did. This indicates that targeting αv integrins in PDGFRβ^+^ precursor cells, rather than α-SMA^+^ terminal cells, may hold greater therapeutic promise. The contribution of immune cells to PF via integrin-mediated pathways remains incompletely understood. Future research should employ co-culture systems to investigate interactions between immune cells and both lung epithelial and fibroblast lineage cells. The repeated failure of integrin inhibitors to translate from preclinical models to clinical efficacy in IPF underscores systemic challenges. We propose that overcoming these hurdles will require advances in target validation, biomarker development, and optimized drug delivery strategies. Currently, the BLM-induced model commonly used in preclinical studies has notable limitations. This model exhibits a self-limiting fibrotic response following acute injury, which fundamentally differs from the chronic, progressive nature of human IPF. Future optimization of this model could include simulating the pathomechanical microenvironment through dynamic matrix stiffness modulation systems, using aged mice or genetically modified models that better reflect human disease progression, and constructing lung-like organoids derived from IPF patients to evaluate the efficacy of integrin inhibitors. Furthermore, existing mechanistic studies are largely confined to phenotypic correlation analyses between integrins and fibrotic progression. However, the validation of causal relationships using gene-editing technologies remains insufficient. While targeting specific integrins such as αvβ6 has shown antifibrotic potential in preclinical models, the effectiveness of single-target inhibition may be undermined by compensatory signaling pathways within the complex pathological microenvironment of IPF. Therefore, the development of more conditional knockout models is essential to elucidate the causal roles of integrins in PF. A combination approach, using integrin inhibitors alongside inhibitors of key downstream signaling molecules, may offer a synergistic strategy to suppress fibrosis more effectively. Clinical trial designs also present challenges. They currently lack a biomarker-based stratification system for integrin expression and fail to implement precise intervention strategies that account for spatiotemporal heterogeneity and intercellular variability. This shortcoming may dilute efficacy signals across heterogeneous patient populations. Elevated αvβ6 integrin levels could serve as a stratification criterion in clinical trials, improving their sensitivity to therapeutic effects [95]. Comparing integrin levels in blood or bronchoalveolar lavage fluid across different ILD types may help identify patients most likely to benefit from integrin-targeted therapies. However, practical challenges in integrating biomarkers into clinical decision-making remain. Standardized workflows for sample collection, isolation, processing, and follow-up testing must be established. Moreover, potential sources of bias in data analysis need to be addressed. The integration of spatial transcriptomics with single-cell sequencing may help identify cellular subtypes with high integrin expression and clarify their functional roles in distinct temporal and spatial contexts, paving the way for novel PF treatments. Future research should also consider targeting integrins such as α4β1, α5β1, α8β1, and α2β2, building on insights from current mechanistic studies. Finally, integrin inhibitors face the challenge of off-target effects. Drugs such as vedolizumab, eptifibatide, and tirofiban have been approved for Crohn’s disease and acute coronary syndromes [70], but their intravenous administration is associated with systemic toxicities, including viral infections [96], hepatotoxicity [97], and increased bleeding risk [98]. In the context of PF, inhalation formulations offer localized delivery to the lungs, improving target specificity and reducing systemic exposure and toxicity. However, inhaler development is technically demanding, requiring precise control of particle size and stability for effective lung deposition. Patient misuse and lower dosing accuracy compared to systemic delivery also present obstacles. Emerging drug delivery systems offer promising solutions due to their enhanced targeting accuracy, improved drug specificity and bioavailability, extended duration of action, and reduced side effects [99]. For instance, a study synthesized and tested three new covalent conjugates combining the small cyclic peptide c(AmpLRGDL), which targets αvβ6 integrins, with the tyrosine kinase inhibitor nintedanib. These conjugates exhibited selective uptake by cells overexpressing αvβ6 and enhanced the antifibrotic effects of nintedanib [100]. Similarly, in liver fibrosis models, bionanoparticles cloaked with hepatic stellate cell (HSC) membranes (HSC-PLGA-BAY) significantly improved delivery of the antifibrotic agent BAY 11-7082 to activated HSCs. This targeting was mediated by homologous adhesion molecules such as integrin αvβ3 and N-cadherin on the cell surface, offering a new direction for integrin-targeted therapy [101].

## Conclusion

By deepening our understanding of integrin specificity across various cell types—their interactions with TGF-β and other signaling pathways, as well as their engagement with the extracellular environment—we aim to translate these insights into innovative therapeutic strategies. Effective integrin-based therapies for PF will require a combination of targeted inhibition, precise patient stratification, and advanced delivery technologies, underscoring the need for balanced, combinatorial approaches that prioritize safety. Future progress will likely depend on detailed analyses of spatiotemporal dynamics within the fibrotic microenvironment and strong interdisciplinary collaboration.
